# Network analysis of autistic disease comorbidities in Chinese children based on ICD-10 codes

**DOI:** 10.1186/s12911-020-01282-z

**Published:** 2020-10-17

**Authors:** Xiaojun Li, Guangjian Liu, Wenxiong Chen, Zhisheng Bi, Huiying Liang

**Affiliations:** 1 Institute of Pediatrics, Guangzhou Women and Children’s Medical Center, Guangzhou Medical University, Guangzhou, 510623 China; 2 Department of Neurology, Guangzhou Women and Children’s Medical Center, Guangzhou Medical University, Guangzhou, 510623 China; 3 School of Basic Medical Sciences, Guangzhou Medical University, Guangzhou, 511436 China

**Keywords:** Autism, Comorbidity, Disease network

## Abstract

**Background:**

Autism is a lifelong disability associated with several comorbidities that confound diagnosis and treatment. A better understanding of these comorbidities would facilitate diagnosis and improve treatments. Our aim was to improve the detection of comorbid diseases associated with autism.

**Methods:**

We used an FP-growth algorithm to retrospectively infer disease associations using 1488 patients with autism treated at the Guangzhou Women and Children’s Medical Center. The disease network was established using Cytoscape 3.7. The rules were internally validated by 10-fold cross-validation. All rules were further verified using the Columbia Open Health Data (COHD) and by literature search.

**Results:**

We found 148 comorbid diseases including intellectual disability, developmental speech disorder, and epilepsy. The network comprised of 76 nodes and 178 directed links. 158 links were confirmed by literature search and 105 links were validated by COHD. Furthermore, we identified 14 links not previously reported.

**Conclusion:**

We demonstrate that the FP-growth algorithm can detect comorbid disease patterns, including novel ones, in patients with autism.

## Background

Autism appearing in infancy and early childhood is a developmental disorder characterized by difficulties with social interaction and communication, and by restricted and repetitive behavior [1]. Autism has become a pressing social concern as the rapid increase in its prevalence has provoked public anxiety [2]. The prevalence of childhood autism is 26.6 per 10,000 people in China [2, 3]. Additionally, it is a lifelong developmental disability and thus autistic patients have a heavy demand for educational, social, and medical services [2]. Consequently, early detection and prevention of autism are urgently needed to reduce the disease burden.

Generally, if the concurrent diseases in patients have a common genesis, they are known as comorbidities [4]. Comorbidities are associated with worse health outcomes, more complex clinical management, and increased health care costs [4]. As a result, comprehensive and individualized treatment needs to be adopted for patients with comorbidities. Therefore, a way to effectively and objectively evaluate comorbidities is imperative for doctors, especially doctors treating patients with autism, as 70% of these patients are reported to have concurrent conditions [5].

Understanding the relationships between comorbid diseases in patients could assist in constructing a disease network (DN) that could provide novel techniques for the diagnosis of comorbid diseases [6]. DNs reveal interrelations between human diseases that share features, such as similarity of symptoms, commonality of etiological environmental factors, and genetic associations [7]. In the last few decades, several network models have been developed to introduce inter-disease linkages. Among them, models based on molecular data were used frequently [7]. Goh et al. [8] built a genetically-based DN, where they showed interconnection between diseases sharing common genes. Moreover, Suthram et al. [9] constructed DNs of diseases sharing mRNA and protein interaction. More recently, studies have constructed DNs based on gene or protein associations via medicine databases such as the Genetic Association Database (GAD) and Online Mendelian Inheritance in Man (OMIM) [10, 11]. Although there has been significant progress in the establishment of DNs using molecular biological data, challenges remain in translating this knowledge to clinical practice [12]. Firstly, DNs based on genetic data cannot explain the relationships between non-genetic disorders. Secondly, significant non-genetic risk factors, such as gender and age, should be considered in clinical diagnostics and treatment; however, previously created genetic DNs are unable to account for these. Moreover, protein and genetic data cannot be applied to clinical practice in real time since the cost of obtaining this data from patients not already tested is too high.

Autism is now considered a complex mental disorder that is associated with a combination of genetic and neurodevelopmental conditions [5, 13]. In this study, we used the International Classification of Diseases 10th Revision (ICD-10) [14] codes to establish a diagnostic DN that can be used in clinical practice to detect comorbid diseases in patients with autism, without the additional costs associated with collecting molecular data.

## Methods

### Data introduction

ICD-10 codes have been widely used in hospital disease diagnosis, as recommended by the World Health Organization. Patients aged ≤ 18 years, with a diagnostic code of F84.0 or F84.1 [14] who were treated at the Guangzhou Women and Children’s Medical Center from 2016 to 2018, were included. Although in the Diagnostic and Statistical Manual of Mental Disorders (DSM-5) the ICD-10 diagnosis code for autism is F84.0, for which the age at diagnosis is less than 3 years [1, 14], the majority of children with autism are diagnosed after 3 years of age [15, 16]. Thus, the ICD-10 code of F84.1, which is autism diagnosed after 3 years of age, was also considered in this study. Furthermore, the following ICD-10 codes were excluded from the DN analysis: V01-Y98 (addition coding) and Z00-Z99 (factors influencing health status and contact with health services), as these codes should not be used for international comparisons [14]. After excluding the aforementioned codes, patients with fewer than two remaining comorbid conditions were excluded from the study. In total, 1488 patients were included in this study. This study was approved by the Ethics Committee and Institutional Review Board of Guangzhou Women’s and Children’s Medical Center, Guangzhou, China, and conducted in accordance with the ethical guidelines of the Declaration of Helsinki of the World Medical Association (IRB No. 2019-06700). The requirement to obtain informed consent was waived because of the retrospective nature of the study. All data were deidentified before they were provided to the investigators.

### Data preprocessing

ICD-10 provides a detailed description of the diseases and conditions, which an interested reader may consult elsewhere. In this study, the focus was on the broad category of autism, rather than the specific characteristics of each subcategory. A detailed classification that contains categories representative of gradation of a broader condition can inflate the rate of false positive results. For example, D64.901 and D64.902 indicate mild anemia and moderate anemia, respectively. However, the broad category of anemia was investigated in this study, as the focus was on disease categories rather than disease gradation. As a result, to ascertain relationships between diseases more accurately, the disease codes were generalized at three levels (Additional file 1: Table 1). Level 0 consisted of original disease codes, which could not be generalized for several reasons. For example, the code F41 represents “other types of anxiety disorders (not otherwise specified, NOS)”. Participants in this study had the F41.001 and F41.101 codes, which indicate different secondary symptoms and thus could not be grouped together. So, they were classified as level 0. Specifically, at level 0 there were four cases in which the original code was retained: (1) The disease codes constituted a three-character category; (2) Diseases with a six-digit subcategory code inclusive of “NOS” or “Not Elsewhere Classified (NEC),” which indicate etiology, anatomic site, severity, or other clinical details; (3) Diseases with codes R90-R94 that represent abnormal findings on examination upon diagnostic imaging or in functional studies; (4) Codes F84.2-F84.9, which were not grouped together with autism (F84). Furthermore, at level 1 there were two cases in which we grouped the codes by their three-character category: (1) The number of three-character categories was greater than one; (2) Codes F84.0 and F84.1 that represented the diagnosis of autism. Level 2 grouping was conducted according to the disease classifications in each chapter of the ICD-10. For example, there were four different codes (F70.900, F71.900, F78.100, and F79.100) for mental retardation. According to Chapter V of the ICD-10, they can be classified as F70–F79 (level 2), thus representing mental retardation as a broad category.

### Disease rules discovery

Association rule mining is considered an effective way to identify potential disease associations. Currently, Apriori and FP-growth are the most commonly used association rule mining algorithms [17]. In this study, the FP-growth algorithm was applied to obtain the comorbid disease pairs since the FP-growth algorithm has higher efficiency than Apriori and a lower requirement for binary computing power [17].

In this algorithm, the support (*sup*) and confidence (*conf*) indicators were used to measure the association rules of interest. However, to discover as many potential rules as possible, lift (*sup*: 0.001, *conf*: 0, *lift:* 1) was used as the main standard for mining association rules. The formulas for the three indicators are given below (Eqs. 1–3) [18]:

1 $$sup\left( {A \Rightarrow B} \right) = P\left( {A \cup B} \right)$$

2 $$conf\left( {A \Rightarrow B} \right) = P\left( {B|A} \right) = sup\_count\left( {A \cup B} \right)/sup\_count\left( A \right)$$

3 $$lift\left( {A,B} \right) = P\left( {A \cup B} \right)/\left( {P\left( A \right)P\left( B \right)} \right)$$

where *Sup* is an indication of how frequently the item set appears in the dataset. The sup of A ⟹ B with respect to dataset T is defined as the proportion of transactions of A ⟹ B in the dataset T which contains the item set A and B. *Conf* is an indication of how often the rule is found to be true. *Conf* of A ⟹ B with respect to the dataset T, is the proportion of the transactions that contains A, which also contains B. *Lift* is the ratio of the observed *sup* to that expected if A and B were independent.

### *k*-fold cross-validation

The *k*-fold cross-validation (*k *= 10) [19] was provided to assess the established disease rules. For clarity, Table 1 shows a hypothesized rule base (Tr = 5) and test case group (n = 3). For P1, when examining disease A, rule 1 and rule 2 are triggered and the prediction is correct. Therefore, a value of 2 for Pt is added to the correct prediction rule set R1. When examining disease B, rule 3 is triggered and the prediction is correct. Thus, a value of 1 for Pt is added to R1. When examining disease C, rule 4 and rule 5 are triggered and the prediction is incorrect. A value of 2 for Pf is added to the incorrect prediction rule set R2. For P1, the predicted diseases are B, C, D, E while the actual diseases P1 has are A, B, C. The disease that has not been predicted is A. Therefore, Prt is 3/3 = 1, Prf = 2/3, and Pn = 1/3. After examining patients P2 and P3, the number of correct predictions in R1 (Nrt) and the number of incorrect predictions in R2 (Nrf) were calculated, as shown in Table 2. For the 10-fold cross-validation, to ensure that the distribution of diseases in each fold is as close to the original distribution as possible, the following grouping rules were applied: firstly, 10 empty case groups are established. Secondly, a disease database was formed by sorting all the diseases in the case set ascendingly according to their frequency. From the patient dataset, the patients diagnosed with the first disease in the disease database were first selected, and the selected patients were then randomly assigned to the 10 case groups. The selected disease and the patients with the disease were then removed from their respective dataset. The same procedure was repeated on the updated disease dataset and patient dataset until no patients was left in the patient dataset. If the cases cannot be distributed evenly, it would be first distributed to the group with fewer cases.

**Table 1:** Assuming rule set and case data

Rule set	A → B	Case	P1	ABC
	A → C			
	B → C		P2	BD
	C → D			
	C → E		P3	ACE

**Table 2:** The results of assuming data

	P1	P2	P3	ALL
Pt	3	0	2	5
Nrt	3	0	2	4
Rt	–	–	–	5/4
Prt	3/3	0/2	2/3	3/3 + 0/2 + 2/3
APrt	–	–	–	(3/3 + 0/2 + 2/3)/3
Pdt	2/3	0/2	2/3	2/3 + 0/2 + 2/3
APdt	–	–	–	(2/3 + 0/2 + 2/3)/3
Pf	2	1	2	5
Nrf	2	1	2	4
Rf	–	–	–	5/4
Prf	2/3	1/2	2/3	2/3 + 1/2 + 2/3
APrf	–	–	–	(2/3 + 1/2 + 2/3)/3
Pdf	2/3	1/2	2/3	2/3 + 1/2 + 2/3
APdf	–	–	–	(2/3 + 1/2 + 2/3)/3
Nrn	–	–	–	0
Pn	1/3	2/2	1/3	1/3 + 2/2 + 1/3
APn	–	–	–	(1/3 + 2/2 + 1/3)/3

The resulting 10 case groups were then used for the 10-fold cross-validation. In the *i*-th cross-validation, the *i*-th group of cases are regarded as the test set; the rest as the training set and the test results of C under different values are respectively calculated. The average and standard deviation of 10-fold cross-validation are obtained.

### Rules verification

To verify the rules, a publicly accessible database, Columbia Open Health Data (COHD), was used. The COHD^1^ is a database derived from the Columbia University Irving Medical Center. The Center’s Observational Health Data Sciences and Informatics database records the EHR (Electronic Health Record) prevalence and comorbid disease frequencies based on pre-existing conditions, drugs, procedures, and demographics (gender, race, and ethnicity) [20]. The database is comprehensive, containing a cross-section of diseases, and covering 36,578 single concepts and 32,788,901 concept pairs from 5,364,781 patients. It also provides direct access to comorbid disease pairs and the results of association analyses between disease pairs (e.g. Chi-square, relative frequency), which allowed us to directly verify the results of this study.

There were two main verification steps used in this study. First, we found the Observational Medical Outcomes Partnership (OMOP) concept ID in the COHD according to the ICD-10 code^2^ (Additional file 1: Table 2). Second, if the disease association rule (disease pair) was in the COHD and the Chi-square analysis in the COHD was significant (*P* < 0.05), the disease association rule was considered true. However, not all disease codes matched the OMOP concept ID exactly. For level 0, fuzzy matching was used to match the ICD-10 code by three or four digits. For example, F41.101 (generalized anxiety disorder) was given priority as F41.1 and F41 when F41.1 was not in the COHD. For level 2, the ICD-10 code was split into the original code to retain three or four digits, and then if any of the disease pairs had significance, the rule was considered true. For example, L20-L30 for which the original codes were L20.800, L30.900, and L30.902, could be split into L20.8 and L30.9 to match the concept ID. If any of the rules including L20.8 and L30.9 were significant in the COHD Chi-square analysis, the rule including L20-L30 was considered true.

The rules were also confirmed by searching the literature since the public databases were incomplete (Additional file 1: Table 3). Generally speaking, autism is one of the terms historically used for autism spectrum disorder (ASD). As a result, the majority of the previously published studies report on ASD, and thus this was considered an autism-representative term in our search. Moreover, although priority was given to pediatric diseases, the adult population was also included in the search.

### Disease network construction

**Fig. 1:**
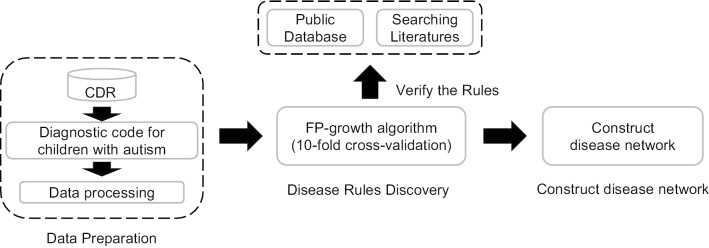
Description of the procedures and steps used in this study. In the data preparation stage, the diagnostic codes for children with autism were extracted from the hospital CDR (Clinical Data Repository) database and were then generalized in the next step. In the disease rule discovery stage, the FP-growth algorithm was first applied to discover all the rules for the relationships between the diseases, and the discovered rules were examined internally using 10-fold cross-validation. All of the rules were further validated by using the public database (COHD) and by searching the literature. Finally, a disease network was constructed using Cytoscape 3.7

After validating the rules using the COHD and relevant literature, the DN was established using Cytoscape 3.7, which is an open source software platform for visualizing complex networks. In the network, the node represents a disease and the link represents the relationship between two diseases by the *conf* indicator. The DN depicted in this study was a directed graph. The same color nodes indicated that the diseases investigated were the same type, based on the chapters in ICD-10. A summary of the overall technical route employed in this study is found in Fig. 1.

## Results

### Basic demographic characteristics 

As shown in Table 3, among 1488 children with autism, males accounted for 82.66% (1230/1488). The children were divided into four age groups: early childhood (0–4 years), middle childhood (5–9 years), early adolescence (10–14 years), and late adolescence (15–18 years). Although patients might have multiple hospital visits within 3 years, the age-group distribution was only calculated for the first visit. Overall, 59.88%, 34.34%, 5.58%, and 0.2% of the first hospital visit occurred in early childhood, middle childhood, early adolescence, and late adolescence, respectively. The majority of patients (96.24%) had one to nine hospital visits. Furthermore, the number of urban patients was significantly higher than rural patients.

**Table 3:** Baseline characteristics of Chinese autistic children

Characteristic	N (%)
Sex	
Male	1230 (82.66%)
Female	258 (17.34%)
Age	
Early childhood	891 (59.88%)
Middle childhood	511 (34.34%)
Early adolescence	83 (5.58%)
Late adolescence	3 (0.20%)
Region^a^	
Urban	1161 (81.76%)
Rural	259 (18.24%)
Visit times	
1–9	1433 (96.24%)
10–19	38 (2.55%)
20-	18 (1.21%)

^a^Data were missing for 68 patients. N = number

### Comorbid diseases in children with autism

**Fig. 2:**
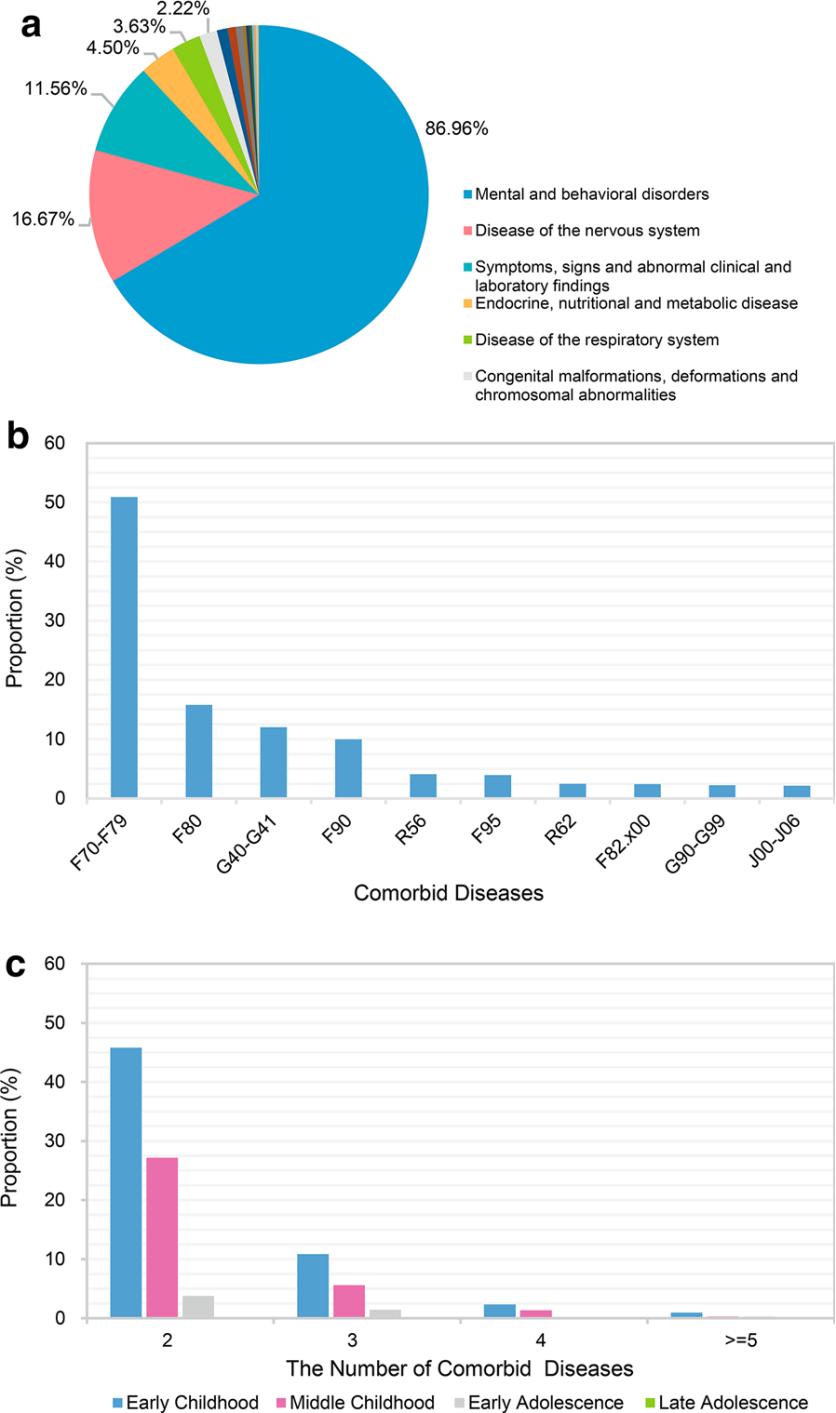
The basic clinical characteristics of the 1488 children with autism included in this study. **a** Distribution of the comorbid disease categories according to the ICD-10 chapter; **b** Top ten comorbid diseases among children with autism; **c** Distribution of the age-group at the first hospital visit based on the number of comorbid diseases. *F70–F79: Intellectual Disability; F80: Developmental Speech Disorder; G40–G41: Epilepsy; F90: Attention Deficit Hyperactivity Disorder; R56: Convulsions; F95: Tic Disorders; R62: Lack of Expected Normal Physiological Development; F82.x00: Developmental Disorder of Motor Function; G90–G99: Other Disorders of the Nervous System; J00–J06: Acute Upper Respiratory Infections

We identified 148 comorbid diseases that occurred among children with autism. There were 17 categories of diseases associated with autism (Fig. 2a). Mental and behavioral disorders (ICD-10, Chapter V) (86.96%) and diseases of the nervous system (ICD-10, Chapter VI) (16.47%) were the leading comorbid disease-categories associated with autism, in addition to non-specific symptoms, signs, and abnormal clinical and laboratory findings (ICD-10, Chapter XVIII). Convulsions (35.47%) and lack of expected normal physiological development (21.51%) were the major symptoms/signs present in children with autism. The most common comorbidities in this group of children were mental retardation (F70–F79), developmental speech disorder (F80), epilepsy (G40–G41), and attention deficit hyperactivity disorder (ADHD, F90) (Fig. 2b). Furthermore, 76.88% of patients had co-occurrence of two diseases, while 23.12% of patients had three or more diseases simultaneously. Most of the children had their first hospital visit at early childhood, irrespective of the number of comorbid diseases (Fig. 2c).

### Disease network

Based on the results of the FP-growth algorithm, a DN with 76 nodes and 178 directed links was constructed in this study. In summary, 59% (105/178) of the links were validated by the COHD; these were considered reliable since the public database has been publicly verified for a long time [20]. On the other hand, 89% (158/178) of the links were confirmed by searching the literature; 99 of these links were also contained in the COHD. Finally, there were only 14 (8%) links which were not validated (Table 4) (Additional file 1: Table 4).

**Table 4:** Agreement of the present study with COHD and literature regarding the identified disease rules

Confirmed by literature	Reported in COHD		Total
	Yes	No	
Yes	99	59	158
No	6	14	20
Total	105	73	178

**Fig. 3:**
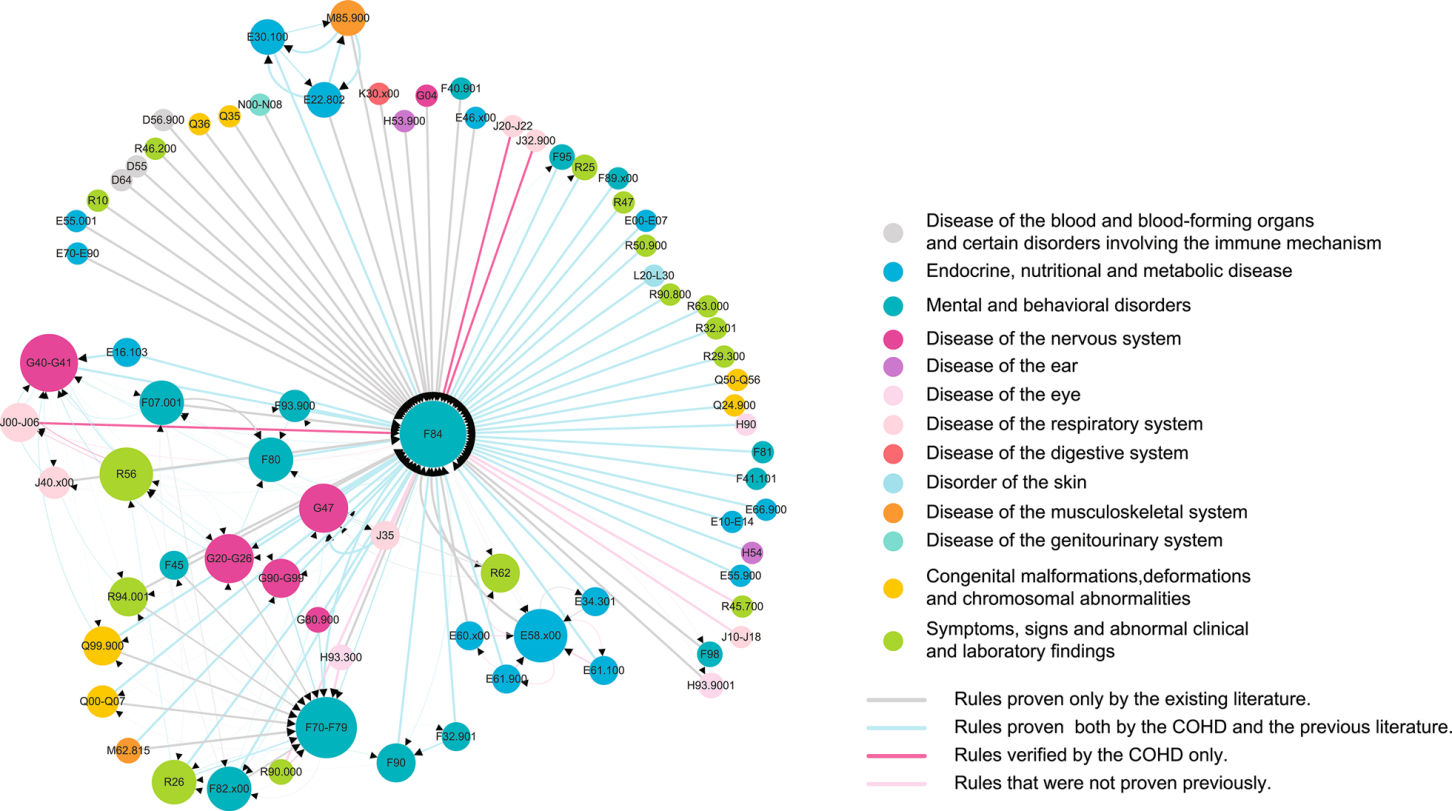
The disease network. One node represents a disease and the size indicates the number of connected lines. A larger node indicates that the node is more important. The link represents the relationship between two diseases by the *conf* indicator. Nodes of the same color indicate same type of disease, while links with different colors represent the different results for the verification of rules

Besides autism (F84), regardless of indegree or outdegree, mental retardation (F70–F79), epilepsy (G40–G41), convulsions (R56), and dietary calcium deficiency (E58.x00) were the biggest nodes in this network (Fig. 3). The common comorbidities included extrapyramidal and movement disorders (G20–G26), sleep disorder (G47), speech disorder (F80), and epilepsy (G40–G41). Moreover, there were 55 (31%) links that were directly connected to autism. Additionally, the distribution of *conf* was not uniform (0.57 ± 0.44). There were 49 (28%) links with *conf* less than 0.1, for which only one link was not proven. The rules for small thresholds were also valid, thus showing the correctness of the threshold selection of the FP-growth algorithm. Further, the DN showed that there were both single nodes, for which the degree was less than or equal to two, as table well as subnetworks. The single nodes were mainly concentrated in the upper left side of the figure, which mainly contained items from “symptoms, signs, and abnormal clinical and laboratory findings” (ICD-10, Chapter XVIII). Additionally, there were approximately four subnetworks in this figure for which the center points were mental retardation (F70–F79), epilepsy (G40–G41), dietary calcium deficiency (E58.x00), and center precocious puberty (E22.802). These subnetworks are mutually contained and not independent of each other; for example, convulsions (R56) were part of a G40–G41-centric subnetwork and F70–F79-centeric subnetwork.

### *k*-fold cross-validation 

As shown in Table 5, Np has a constant value of 148.8. When C is 0, Pt is significantly larger than Np, which indicates that on average, the diseases of each patient have been correctly predicted many times. When comparing Nrt and Tr, we found that over 44.40% of the rules have produced correct predictions. The Rt value is larger than 1, indicating that the rules are used repeatedly, i.e. the rules have a certain degree of universality. APrt is larger than 1, indicating that the average number of the correct predictions per patient is larger than the actual number of the diseases they have. The rule set has a certain degree of redundancy, and there exists a possibility that a disease might be correctly predicted for multiple times. APdt is high, with an average of 93.16% of diseases being correctly predicted. It also shows that the prediction accuracy of this rule set is relatively high.

**Table 5:** The results of 10-fold cross-validation

	C	Tr	Correct predictions						Incorrect predictions					Rules untriggered	
			Pt	Nrt	Rt	Np	APrt	APdt	Pf	Nrf	Rf	APrf	APdf	Nrn	APn
Avg (Std)	0	164.6 (4.0)	381.4 (14.8)	73.0 (3.9)	5.23 (0.2)	148.8 (0.8)	1.06 (0.02)	0.93 (0.01)	5714.0 (197.9)	105.7 (4.5)	54.16 (3.24)	15.52 (0.26)	0.95 (0.02)	51.2 (3.6)	0.07 (0.01)
	0.1	118.7 (3.0)	330.8 (11.2)	47.5 (2.5)	6.98 (0.3)	148.8 (0.8)	0.94 (0.01)	0.84 (0.01)	884.0 (52.7)	59.8 (3.5)	14.83 (1.31)	2.25 (0.05)	0.65 (0.01)	51.2 (3.6)	0.16 (0.01)
	0.2	102.6 (2.5)	271.0 (10.1)	42.0 (3.1)	6.47 (0.4)	148.8 (0.8)	0.77 (0.01)	0.67 (0.01)	286.3 (35.5)	43.8 (4.0)	6.57 (0.85)	0.63 (0.04)	0.42 (0.01)	51.1 (3.4)	0.33 (0.01)
	0.3	95.1 (3.0)	270.3 (9.9)	41.3 (2.4)	6.56 (0.3)	148.8 (0.8)	0.76 (0.01)	0.67 (0.01)	268.1 (33.3)	37.1 (3.0)	7.28 (1.11)	0.59 (0.03)	0.41 (0.01)	50.3 (3.1)	0.33 (0.01)
	0.4	93.4 (3.1)	268.1 (9.0)	40.3 (2.4)	6.67 (0.3)	148.8 (0.8)	0.76 (0.01)	0.67 (0.00)	256.0 (29.3)	35.8 (3.0)	7.20 (1.02)	0.57 (0.03)	0.40 (0.01)	49.9 (3.0)	0.33 (0.00)
	0.5	92.5 (3.3)	268.1 (9.0)	40.3 (2.4)	6.67 (0.3)	148.8 (0.8)	0.76 (0.01)	0.67 (0.00)	255.4 (29.5)	35.4 (2.9)	7.26 (1.02)	0.57 (0.03)	0.40 (0.01)	49.4 (3.2)	0.33 (0.00)
	0.6	88.2 (2.8)	190.4 (6.3)	37.9 (2.0)	5.03 (0.2)	148.8 (0.8)	0.53 (0.01)	0.46 (0.00)	131.7 (25.6)	32.4 (2.9)	4.09 (0.85)	0.24 (0.03)	0.18 (0.02)	48.1 (2.6)	0.54 (0.00)
	0.7	80.8 (2.6)	185.7 (6.0)	34.9 (2.0)	5.33 (0.2)	148.8 (0.8)	0.52 (0.01)	0.45 (0.01)	120.2 (26.3)	28.7 (2.7)	4.21 (0.90)	0.22 (0.03)	0.17 (0.01)	44.4 (2.4)	0.55 (0.01)
	0.8	79.2 (2.7)	184.2 (5.5)	34.0 (1.9)	5.43 (0.2)	148.8 (0.8)	0.52 (0.01)	0.44 (0.01)	114.2 (24.9)	27.1 (2.7)	4.24 (0.94)	0.20 (0.03)	0.16 (0.01)	44.4 (2.4)	0.56 (0.01)
	0.9	77.2 (2.3)	182.7 (5.3)	32.5 (1.7)	5.63 (0.2)	148.8 (0.8)	0.52 (0.01)	0.44 (0.01)	111.0 (23.6)	25.4 (2.8)	4.41 (0.97)	0.20 (0.03)	0.15 (0.01)	44.1 (2.2)	0.56 (0.01)
	1	77.2 (2.3)	182.7 (5.3)	32.5 (1.7)	5.63 (0.2)	148.8 (0.8)	0.52 (0.01)	0.44 (0.01)	111.0 (23.6)	25.4 (2.8)	4.41 (0.97)	0.20 (0.03)	0.15 (0.01)	44.1 (2.2)	0.56 (0.01)

However, Pf is as high as 5714 at this time. Nfr is 105.7, which accounts for 64.22% of the total number of rules. Rf, APrf, and APdf are relatively higher. On average, each rule with incorrect predictions is triggered more than 54 times and the number of incorrect predictions is 15 times the actual number of diseases of the patients. The number of incorrectly predicted diseases is almost the same as the actual number of diseases of the patients. There are two reasons for this: First, the *conf*  threshold of the rule set is too low at the time and there is a large number of low-quality rules. Second, by examining Tr, Nrt, Nrf, and Nrn, it shows that an average of 65.3 rules yielded both correct and incorrect predictions at the same time, accounting for 39.67% of the total number of rules. It indicates that these rules only reflect the common characteristics of some patients, and that patients may be grouped based on certain rules, which may lead to a more targeted analysis. On average, 6.84% of diseases have not been correctly predicted, and most of these diseases are very rare in the dataset, with some diseases only appearing once or twice such as cataract, and therefore the corresponding rules cannot be generated.

As C increased to 0.1, Tr decreased significantly, and most of the other indicators decreased as well, especially Pf, which decreased by more than 80%. However, Rt increased significantly. This indicates that the rules eliminated by the increase in the value of C are the rules with lower prediction accuracy and higher incorrect prediction rate. These rules do not have a high universality in the test data. APn also showed a large increase, indicating that the coverage of the rule set has shrunk after the rule is eliminated.

The further increase of the value of C shows that the pattern of change is basically consistent with the above-mentioned description. Although individual data occasionally showed depicted small fluctuations contrary to the above-mentioned pattern, the overall trend remained the same. The change of C in [0, 0.2] and [0.5, 0.6] led to a significant change in the remaining data. When C is 0.3, Rf showed obviously abnormal changes, and the reason is unknown. Thus, the value of C might have a greater impact on the clinical application of this method.

## Discussion

### The rules verified by COHD

According to the rules verified by the COHD, mental retardation, epilepsy, and sleep disorder were commonly associated with autism, as is consistent with the findings from previous studies [1, 20–22]. However, while some comorbidities associated with autism have been reported in the literature, they are frequently ignored in clinical practice due to lack of attention and/or insignificant clinical manifestations. These comorbid diseases include dermatitis and eczema (L20–L30), disorders of the thyroid gland (E00–E07), and fever (R50.900). It has been reported that atopic dermatitis, also known as atopic eczema, has been linked to more specific mental health disorders, which may increase the risk of mental illness in children, including ADHD, depression, and autism [23, 24]. Conversely, the behavior of children with autism appears to be related to thyroid function; however, how thyroid hormones alter behavior and cognition in autism is unclear [25]. Additionally, some children with autism show improved behavior when they have fever because fever may play a role in the severity of autistic symptoms [26]. The ameliorating effects of fever on autistic behaviors may involve interactions between systemic immune responses and neurobiological mechanisms of cortical and neuronal function [27].

Overall, there were four rules directly connected with autism in the COHD which have not been confirmed by previously published literature:

1. chronic sinusitis (J32.900) ⟹ autism (F84).
2. acute lower respiratory infections (J20–J22) ⟹ autism (F84).
3. acute upper respiratory infection (J00–J06) ⟹ autism (F84).
4. autism (F84) ⟹ acute upper respiratory infection (J00–J06).

For example, acute bronchitis infections, which are a type of acute lower respiratory infections, and immune aberrations, have both been reported in autistic children [28]; a dysregulated immune response could increase susceptibility to viral or bacterial infections. Although the role of viruses in autism is unclear, previous hypotheses have linked autism to viral infections, which may become active as a result of defects in individuals’ immune systems [29]. Additionally, as more than 90% of acute bronchitis infections are caused by viruses [30], it follows that autism might be associated with acute bronchitis infections.

### Rules confirmed by the literature

In the networks, there were 158 rules confirmed by the literature; 37% (59/158) of the rules were not verified by the COHD. For example, R94.001 (Electroencephalography, EEG) was confirmed by the literature search but not by the COHD. Previous research has shown that not all children with autism have abnormal EEGs. Tuchman and Rapin [31] examined the EEGs of 392 children with autism with or without a history of epilepsy. The EEGs were epileptiform in 59% of children with autism with histories of epilepsy, while they were such in only 8% of the 335 children without a history of seizures. Seizures are common in ASD, occurring in 20–30% of children with ASD [32]. Generally, the relationships between autism, epilepsy, and abnormal EEGs remain unclear. The association between abnormal EEGs and autism was excluded in the COHD, because the public database is incomplete.

### Unproved rules

**Fig. 4:**
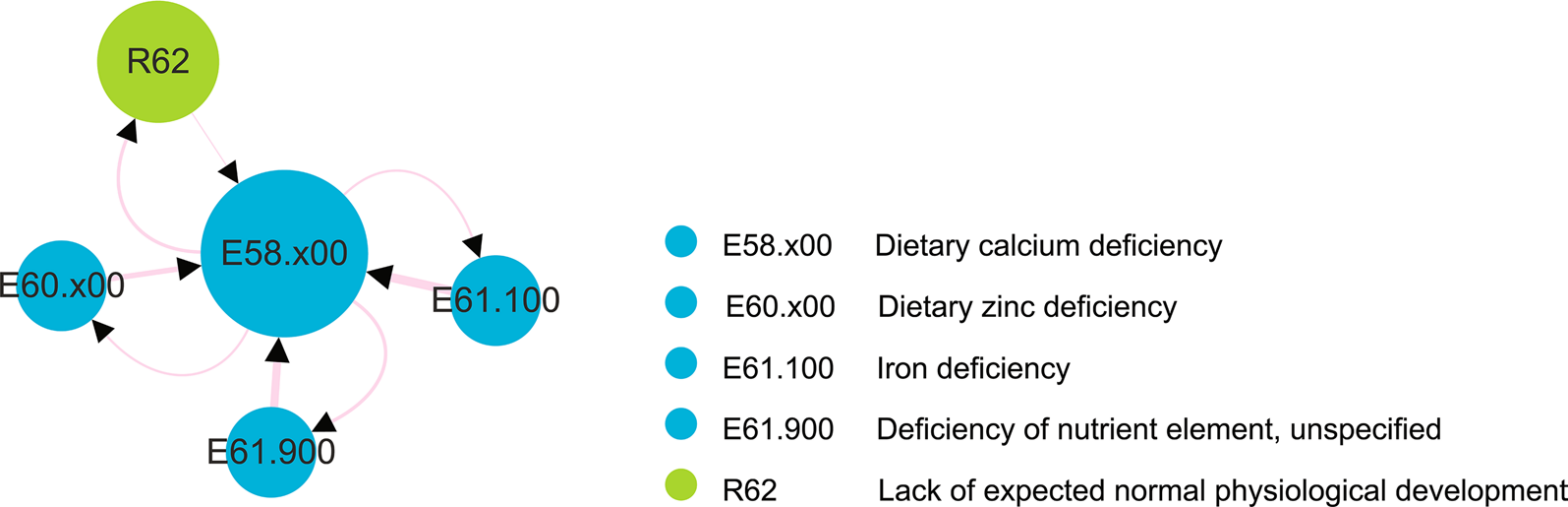
Unverified rules describe a link among nutrient deficiencies in children with autism

The present study discovered 14 (8%) new association rules that have not been reported in the literature or in the COHD, but might be informative and useful. Among these 14 new rules, 8 were associated with nutrient intake, including iron, calcium, and zinc (Fig. 4). The strength of these associations (*conf*) was 0.59 ± 0.35.

Feeding disorders are often reported in children with autism [33–35], with food selectivity being the most common eating disorder [33, 36]. Among children with autism, food selectivity often encompasses strong preferences for carbohydrates, snacks, and/or processed foods, while rejecting fruits and vegetables [37]. Additionally, a growing number of studies implicate the microbiome-gut brain axis in autistic etiology. Since 50% of microbial changes are attributable to diet, these findings strongly support a relationship between autism and nutrition [33]. Moreover, long-term malnutrition can easily impede normal physiological development. In this study, dietary calcium deficiency (E58.x00), iron deficiency (E61.100), and dietary zinc deficiency (E60.x00) were the main types of deficiencies seen in children with autism. Calcium, iron, and zinc are essential microelements important for maintaining normal physiological functions of the body. Together, these results suggest a plausible link between autism and nutrient deficiencies.

## Conclusion

This study has several limitations. Although the validation through data contained in a public database was introduced to reduce the workload and prevent the association rule mining algorithm from producing meaningless rules, it also eliminated some meaningful rules reported in the literature, such as between abnormal EEGs and autism. While a literature search can be an effective validation method, it is time-consuming. Thus, relying on public databases for initial verification reduces workload, and any unvalidated rules can be further verified through literature searches. Moreover, some of the comorbidities documented in previous studies were not found in this study, such as gastrointestinal symptoms in autism. This might be due to the fact that these symptoms are not the primary concern of visiting patients and thus are not reported in medical records, and/or the fact that diagnostic ICD-10 codes, which are singular data points, are not comprehensive enough to capture complex data. Therefore, in order to explore more disease associations, we intend to mine the rest of the data contained in EHRs, such as medical textual records, medications and test results [38, 39].

In clinical practice, comorbid diseases are a common phenomenon. We combined a FP-growth algorithm with networks to discover existing and potential associations between diseases, which can potentially help better understand the co-occurrence between high-risk diseases. Significantly, among all the disease association rules in the network, some of which have been well documented in a public database (59%) and others confirmed based on the findings from previous studies (89%), only 8% of the rules have not yet been validated. In the network, there were 49 (28%) links with *conf* less than 0.1, for which only one link was not validated. It also showed the correctness of the threshold selection for the FP-growth algorithm. Moreover, the validated rules suggest that the methods used in this study can effectively determine disease associations, which provide important insights regarding the detection of patterns of comorbid diseases. Additionally, if applied in clinical practice, DNs can play a role in supporting clinicians in effectively evaluating the risk of particular comorbidities. Furthermore, identifying new rules through DNs suggests that this method has the ability to discover disease associations that have not been previously described. Although association rule mining cannot detect the causality between diseases, novel associations found in this way may be followed up over time to further examine the relationships between diseases and physical symptoms.

## Supplementary information

**Additional file 1.** **Table 1**. Disease generalization in ICD-10 codes. **Table 2**. Comparison among OMOP ID, Concept Code and the generlization ICD-10 codes. **Table 3**. The rules verified by literatures. **Table 4**. The rules discoveried by FP-growth algorithm.

## Data Availability

All data analyzed during this study are included in this published article and its additional files.

## Abbreviations

ICD-10: International Classification of Diseases 10th Revision
COHD: Columbia Open Health Data
DN: Disease network
NOS: Not otherwise specified
Sup: Support
Conf: Confidence
EHR: Electronic health record
ASD: Autism spectrum disorder
ADHD: Attention deficit hyperactivity disorder
EEG: Electroencephalography
C: Confidence threshold
Np: The number of patients
Tr: The total number of rules in the rule set, which decreases as the confidence threshold increases
Pt: The number of correct predictions using the rules
Nrt: The number of rules with correct prediction results
Rt: The number of times the disease is correctly predicted divided by the number of rules that produce correct predictions, namely Pt/Nrt
Prt: The number of rules with correct prediction rules (Pt) divided by the number of the diseases the patient has
APrt: The average value of Prt
Pdt: The number of disease that correctly predicted divided by the number of diseases the patient has
APdt: The average value of Pdt
Pf: The number of incorrect predictions using the rules
Nrf: The number of rules that predict incorrectly
Rf: The number of incorrect predictions using the rules divided by the number of rules that predict incorrectly, namely Pf/Nrf
Prf: The number of incorrect predictions using the rules (Pf) divided by the number of diseases the patient has
APrf: The average value of Prf
Pdf: The number of each patient’s disease incorrectly predicted divided by the number of the diseases the patient has
APdf: The average value of Pdf
Nrn: The number of rules not triggered
Pn: The number of patients with unpredicted diseases divided by the number of the diseases the patient has
APn: The average value of Pn

## Footnotes

1 https://cohd.io/.

2 https://athena.ohdsi.org/search-terms/terms.

## Software and code

In this study, Weka^3^ was used for disease rules discovery and 10-fold cross-validation. And the DN was established using Cytoscape 3.7.^4^
